# Differential diagnosis for suspected cases of coronavirus disease 2019: a retrospective study

**DOI:** 10.1186/s12879-020-05383-y

**Published:** 2020-09-18

**Authors:** Qiong Chi, Xinjian Dai, Xiangao Jiang, Lefei Zhu, Junyan Du, Yuxi Chen, Jiyang Zheng, Jianping Huang

**Affiliations:** 1 Department of Respiratory and Critical Care Medicine, Wenzhou Central Hospital, Wenzhou, Zhejiang China; 2 Department of Infectious Diseases, Wenzhou Central Hospital, Wenzhou, Zhejiang China; 3 Department of Endocrinology, Wenzhou Central Hospital, Wenzhou, Zhejiang China; 4 Department of Gastroenterology, Wenzhou Central Hospital, Wenzhou, Zhejiang China; 5 Department of Emergency, Wenzhou Central Hospital, Wenzhou, Zhejiang China; 6 Department of Neurology, Wenzhou Central Hospital, Wenzhou, Zhejiang China

**Keywords:** COVID-19, Differential diagnosis, Suspected cases, Confirmed cases

## Abstract

**Background:**

Since December 2019, the coronavirus disease 2019 (COVID-19) has infected more than 12,322,000 people and killed over 556,000 people worldwide. However, Differential diagnosis remains difficult for suspected cases of COVID-19 and need to be improved to reduce misdiagnosis.

**Methods:**

Sixty-eight cases of suspected COVID-19 treated in Wenzhou Central Hospital from January 21 to February 20, 2020 were divided into confirmed and COVID-19-negative groups based on the results of real-time reverse transcriptase polymerase chain reaction (RT-PCR) nucleic acid testing of the novel coronavirus in throat swab specimens to compare the clinical symptoms and laboratory and imaging results between the groups.

**Results:**

Among suspected patients, 17 were confirmed to COVID-19-positive group and 51 were distinguished to COVID-19-negative group. Patients with reduced white blood cell (WBC) count were more common in the COVID-19-positive group than in the COVID-19-negative group (29.4% vs 3.9%, *P* = 0.003). Subsequently, correlation analysis indicated that there was a significant inverse correlation existed between WBC count and temperature in the COVID-19-positive patients (*r* = − 0.587, *P* = 0.003), instead of the COVID-19-negative group. But reduced lymphocyte count was no different between the two groups (47.1% vs 25.5%, *P* = 0.096). More common chest imaging characteristics of the confirmed COVID-19 cases by high-resolution computed tomography (HRCT) included ground-glass opacities (GGOs), multiple patchy shadows, and consolidation with bilateral involvement than COVID-19-negative group (82.4% vs 31.4%, *P* = 0.0002; 41.2% vs 17.6% vs *P* = 0.048; 76.5% vs 43.1%, *P* = 0.017; respectively). The rate of clustered infection was higher in COVID-19-positive group than COVID-19-negative group (64.7% vs 7.8%, *P* = 0.001). Through multiplex PCR nucleic acid testing, 2 cases of influenza A, 3 cases of influenza B, 2 cases of adenovirus, 2 cases of *Chlamydia pneumonia*, and 7 cases of *Mycoplasma pneumoniae* were diagnosed in the COVID-19-negative group.

**Conclusions:**

WBC count inversely correlated with the severity of fever, GGOs, multiple patchy shadows, and consolidation in chest HRCT and clustered infection are common but not specific features in the confirmed COVID-19 group. Multiplex PCR nucleic acid testing helped differential diagnosis for suspected COVID-19 cases.

## Background

Since December 2019, the epidemic of pneumonia caused by novel coronavirus in China, has continued to progress [1], having now infected more than 12,322,000 people and killed over 556,000 people worldwide [2]. On February 11, 2020, The International Committee on Taxonomy of Viruses officially named this severe acute respiratory syndrome coronavirus 2 (SARS-CoV-2) and the World Health Organization (WHO) named the disease coronavirus disease 2019 (COVID-19) [3]. Phylogenetic analysis revealed that SARS-CoV-2 falls into the genus betacoronavirus, which includes coronaviruses (SARS-CoV, bat SARS-like CoV, and others) discovered in humans, bats, and other wild animals [4]. On March 11, 2020, the WHO also designated COVID-19 a pandemic [2]. According to epidemiological investigations, the general population is susceptible to SARS-CoV-2, which has the possible route of transmission via droplets, fecal matter, and contact [5]. Because symptoms overlap significantly with other respiratory infections like influenza, diagnosis remains difficult.

Wenzhou had hundreds of confirmed imported cases of COVID-19 and even more suspected cases. Measures to more rapidly and accurately diagnose suspected cases of COVID-19 are challenges that urgently need to be addressed by clinicians. We therefore conducted this study to investigate the clinical characteristics of suspected cases of COVID-19 and to improve the differential diagnosis for COVID-19, thus reduce misdiagnosis.

## Methods

### Patients and data collection

We retrospectively collected the clinical data, including demographics, clinical manifestations, laboratory and radiological findings and contact history of suspected COVID-19 cases in isolation ward (single rooms) of Wenzhou Central Hospital from January 19, 2020 to February 20, 2020. The diagnostic criteria [6] of suspected cases were: individuals matching any one of the criteria for epidemiological history and any 2 of the clinical manifestations, or individuals matching any 3 of the clinical manifestations when there was no definitive epidemiological history. Epidemiological history included (1) history of travel or residence in Wuhan within 14 days before the disease onset; (2) history of contact with patients confirmed with COVID-19 within 14 days before the disease onset; (3) history of contact with individuals with respiratory symptoms who came from Wuhan or communities with reported COVID-19 cases within 14 days before the disease onset; and (4) clustered disease, meaning ≥2 cases with fever and/or respiratory symptoms. Clinical manifestations included (1) fever, (2) chest imaging showing multiple small patchy shadows and interstitial changes, particularly in the lung periphery, during the early stages, which progressed to multiple ground-glass opacities (GGOs), infiltrates, and consolidation; and (3) normal or reduced total white blood cell (WBC) count or reduced lymphocyte count in the early stages. Criteria to confirm or rule out the diagnosis of COVID-19 was as follows [6]: Confirmed COVID-19 cases: positive real-time reverse transcriptase polymerase chain reaction (RT-PCR) SARS-CoV-2 nucleic acid testing or viral gene sequencing showing high sequence homology to known gene sequences of SARS-CoV-2; COVID-19-negative cases: suspected cases with 2 consecutive negative results of respiratory pathogen nucleic acid testing (sampling time interval at least 1 day). In the same period, these samples were analyzed by multiplex PCR named GeXP assay (multiplex PCR combined with automated capillary electrophoresis) for 13 common respiratory pathogens including Influenza A (Flu A), Influenza B (Flu B), Influenza A H1N1 pdm09 (09H1), influenza H3N2 (H3), human para-influenza virus (HPIV), respiratory syncytial virus (RSV), rhinovirus (HRV), adenovirus (ADV), human metapneumovirus (HMPV), human bocavirus (HBoV), human coronavirus (HCoV), Chlamydia (Ch) and *Mycoplasma pneumonia*e (Mp) [7].

### Statistical analysis

Characteristics of patients were summarized using descriptive statistics. The continuous variables were presented as mean ± standard deviation (Mean ± SD), and the comparison between groups was analyzed by independent sample t-test. Categorical variables were expressed as the counts and percentages of patients in each category, and the group comparisons were performed using Chi-square test or Fisher’s Exact test or Chi-square. *P* < 0.05 was considered statistically significant. The SPSS 22.0 software (IBM SPSS Inc., Chicago, IL) was used for statistical analysis in this study.

## Results

Sixty-eight suspected COVID-19 cases were recruited retrospectively to our study from January 21 to February 20, 2020. Among them, 17 were confirmed to be COVID-19 positive and 51 were COVID-19 negative. The clinical symptoms were no difference between two groups (Table 1).

**Table 1:** Demographics and clinical manifestations of suspected cases of COVID-19

	Confirmed COVID-19 group *n* = 17	COVID-19-negative group *n* = 51	*P* value
Male, n (%)	9 (52.9%)	34 (66.7%)	0.309
Age, mean (SD),year	53.5 (13.4)	41.3 (17.9)	0.012
**Medical history, n (%)**			
Hypertension	2 (11.8%)	5 (9.8%)	0.818
Diabetes	1 (5.9%)	2 (3.9%)	NA
Coronary heart disease	1 (5.9%)	2 (3.9%)	NA
Chronic pulmonary diseases	0	2 (3.9%)	NA
Malignant tumors	0	1 (2.0%)	NA
Epidemiological history	11 (64.7%)	6 (11.8%)	0
**Clustered infection, n (%)**	11 (64.7%)	4 (7.8%)	< 0.001
Familial clustering	6 (35.3%)	2 (3.9%)	0.001
shopping center Clustering	5 (29.4%)	2 (3.9%)	0.003
**Clinical manifestations, n (%)**			
Fever	14 (82.4%)	38 (74.5%)	0.509
37–38 °C	6 (35.3%)	19 (37.3%)	0.885
38–39 °C	5 (29.4%)	14 (27.5%)	0.876
≥ 39 °C	3 (17.6%)	5 (9.8%)	0.385
Cough	12 (70.6%)	27 (52.9%)	0.203
Fatigue	7 (41.2%)	12 (23.5%)	0.160
Expectoration	5 (29.4%)	13 (25.5%)	0.751
Others			
Sore through	3 (17.6%)	5 (9.8%)	0.385
Intolerance of cold	3 (17.6%)	9 (17.6%)	1.0
Chest tightness	2 (11.8%)	5 (9.8%)	0.818
Dyspnea	2 (11.8%)	3 (5.9%)	0.421
Palpitations	1 (5.9%)	3 (5.9%)	1.0
Diarrhea	1 (5.9%)	2 (3.9%)	NA
Nausea and vomiting	1 (5.9%)	2 (3.9%)	NA
Hemoptysis	0	2 (3.9%)	NA

*COVID-19* coronavirus disease 2019, *NA* not applicable

**Fig. 1:**
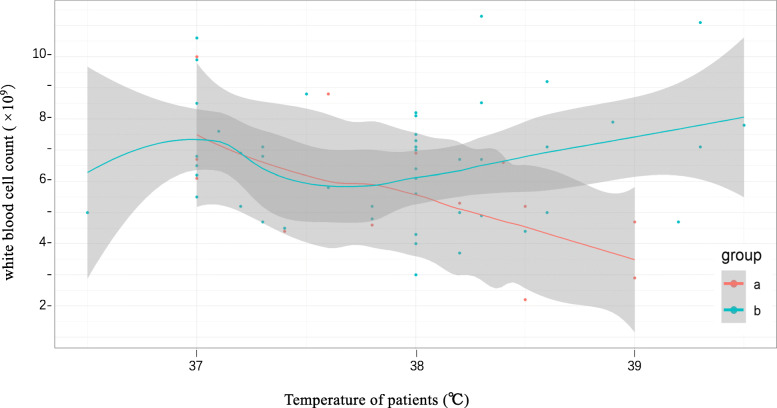
catter plot of temperature and white blood cell count in suspected coronavirus disease 2019 (COVID-19) patients. **a** Patients of the confirmed COVID-19 group; **b** patients of the COVID-19-negative group

**Fig. 2:**
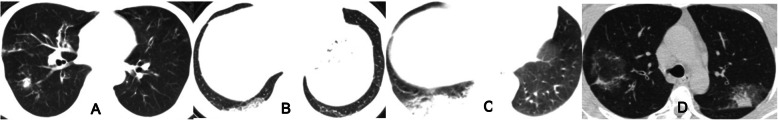
Chest computed tomography (CT) images of pneumonia caused by other pathogens in the coronavirus disease 2019 (COVID-19)-negative group. **a** Pneumonia caused by influenza A virus: scattered and patchy shadows and nodular shadows, with some of the nodular shadows surrounding the bronchovascular bundles; **b** pneumonia caused by influenza B virus: subpleural patchy shadows in the right lower lung; **c** pneumonia caused by adenovirus: consolidation near pleura of the right lower lung; and **d** Chlamydia pneumonia: multiple ground-glass opacities (GGOs) and consolidations in both lungs

**Fig. 3:**
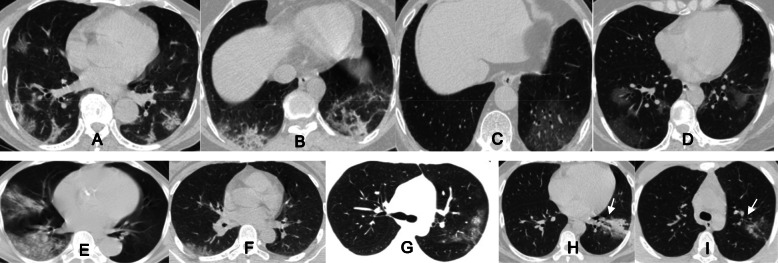
Chest computed tomography (CT) of patients in the 4 familial clusters. **a & b** The first familial cluster of coronavirus disease 2019 (COVID-19). The chest CT of the husband and wife showed bilateral patchy shadows and grid-like interstitial change in the lower lobes and; **c & d** The second familial cluster of COVID-19. The chest CT showed a single ground-glass opacity (GGO) in the left lower lung near the pleura of the husband and multiple GGO near pleura in both lungs of the wife. **e & f** The third familial cluster of COVID-19. The chest CT showed multiple GGOs and consolidation near the pleural of the right lung of the husband and patchy shadows near the pleura in the right lung in the wife. **g** The son in the third familial cluster had multiple GGOs and patchy shadows in the left lung but the diagnosis could not be confirmed. **h & i** The fourth familial cluster of *Mycoplasma pneumonia*. Both father and son patients had centrilobular nodules, GGOs, consolidation together with bronchial wall thickening (indicated by arrows)

Laboratory tests, chest imaging, and nucleic acid testing are shown in Table 2. Patients with reduced WBC count were more common in the confirmed COVID-19 group than in the COVID-19-negative group (29.4% vs 3.9%, *P* = 0.003). Subsequently, correlation analysis indicated that there was a significant inverse correlation existed between WBC count and temperature in the COVID-19-positive patients (*r* = − 0.587, *P* = 0.003), instead of the COVID-19-negative group (Fig. 1). But reduced lymphocyte count was not found to be significantly different between the two groups (47.1% vs 25.5%, *P* = 0.096). More common chest imaging characteristics of the confirmed COVID-19 cases by high-resolution computed tomography (HRCT) included GGOs, multiple patchy shadows, and consolidation with bilateral involvement than COVID-19-negative group (82.4% vs 31.4%, *P* = 0.0002; 41.2% vs 17.6% vs *P* = 0.048; 76.5% vs 43.1%, *P* = 0.017; respectively). Bronchial wall thickening (9.8%) and reversed halo signs (2.0%) only saw in Chest HRCT of the COVID-19-negative group. 13 (76.5%) SARS-CoV-2 nucleic positive were identified in the first test of RT-PCR. 17.6% patients appeared negative results in the first round of test but turned to positive in the second round of test. Among the COVID-19-negative cases, 1 patient (2.0%) had a weakly positive result in the first viral nucleic acid test, but had negative results in the two following re-tests. Among the patients in the COVID-19-negative group, multiplex PCR testing showed 2 (3.9%) cases of influenza A with characteristic scattered and patchy shadows and nodular shadows in both lungs (Fig. 2a), 3 (5.9%) cases of influenza B with characteristic subpleural patchy shadows in chest CT (Fig. 2b), 2(3.9%) cases of adenovirus with characteristic consolidation near the pleura in chest CT (Fig. 2c), 2(3.9%) cases of *Chlamydia pneumoniae with* characteristic multiple GGOs and consolidations in both lungs (Fig. 2d), and 7(13.7%) cases of *Mycoplasma pneumoniae* infections with characteristic bronchial wall thickening, centrilobular nodules, GGOs and consolidation in Chest HRCT (Fig. 3h & i). No co-infections was observed in the COVID-19-positive or COVID-19-negative patients.

**Table 2:** Laboratory tests, chest imaging, and nucleic acid testing of suspected cases of COVID-19

	Confirmed COVID-19 group *n* = 17	COVID-19-negative group *n* = 51	*P* value
White blood cell count, mean (SD), ×10^9^/L	5.27 ± 2.08	6.73 ± 1.94	0.010
< 4, n (%)	5 (29.4%)	2 (3.9%)	0.003
4–10, n (%)	12 (70.6%)	47 (92.2%)	0.023
> 10, n (%)	0	2 (3.9%)	NA
Lymphocyte count, mean (SD), ×10^9^/L	1.35 ± 0.83	1.63 ± 0.81	0.224
< 1.1, n (%)	8 (47.1%)	13 (25.5%)	0.096
≥ 1.1, n (%)	9 (52.9%)	38 (74.5%)	
Hemoglobin concentration, mean (SD), (g//L)	135.18 ± 17.02	138.66 ± 20.33	0.528
< 120, n (%)	3 (17.6%)	7 (13.7%)	0.693
≥ 120, n (%)	14 (82.4%)	44 (86.3%)	
Platelet count, mean (SD), (×10^9^/L)	198.41 ± 92.02	217.52 ± 72.42	0.384
< 100, n (%)	1 (5.9%)	2 (2.9%)	NA
≥ 100, n (%)	16 (94.1%)	49 (96.1%)	
C-reactive protein, mean (SD), (mg/L)	29.27 ± 31.30	17.25 ± 23.31	0.097
**Chest CT, n (%)**			
Ground-glass opacities	14 (82.4%)	16 (31.4%)	0.0002
Consolidation	7 (41.2%)	9 (17.6%)	0.048
Patchy shadows	13 (76.5%)	22 (43.1%)	0.017
Grid-like images	3 (17.6%)	2 (3.9%)	
Bronchial wall thickening	0	5 (9.8)	NA
Reversed halo sign	0	1 (2.0)	NA
Bilateral pulmonary involvement	14 (82.4%)	8 (15.7%)	< 0.001
**SARS-CoV-2 nucleic acid testing, n (%)**			
Positive in the first test	13 (76.5%)	0	
Positive in the second test	3 (17.6%)	0	
Weak positive in the first test	1 (5.9%)	0	
Suspected positive in the first test	1 (5.9%)	1 (2.0%)	
**Multiplex PCR, n (%)**			
Influenza A	0	2 (3.9%)	
Influenza B	0	3 (5.9%)	
Adenovirus	0	2 (3.9%)	
Chlamydia pneumoniae	0	2 (3.9%)	
*Mycoplasma pneumonia*e	0	7 (13.7%)	

*COVID-19* coronavirus disease 2019, *CT* computed tomography, *NA* not applicable, *RT-PCR* reverse transcriptase polymerase chain reaction, *SARS-CoV-2* severe acute respiratory syndrome coronavirus

The rate of clustered infection was higher in COVID-19-positive group than COVID-19-negative group (64.7% vs 7.8%, *P* = 0.001). The first familial cluster of COVID-19 involved transmission from a wife (who visited a physician due to 10 days of fever; she was confirmed to be SARS-CoV-2 positive in the second round of nucleic acid testing) to her husband (fatigue for a week and a day of fever; positive result on first SARS-CoV-2 nucleic acid test). Chest HRCTs showed a grid images in the inferior lobes of both lungs, especially obvious in the lung periphery (Fig. 3a & b). In the second cluster, the wife had a positive result in the first SARS-CoV-2 nucleic acid testing after 15 days of fever and her chest computed tomography (CT) showed multiple GGOs near the bilateral pleura. Her husband had a negative result in the first SARS-CoV-2 nucleic acid testing but a positive result upon the re-test on the first day of his fever and his chest CT showed a single GGO in the left lower lung near the pleura (Fig. 3c & d). The third familial cluster involved transmission from husband (fever and cough for 13 days; positive result in the first SARS-CoV-2 nucleic acid testing) to his wife who remained asymptomatic (weak positive result in the first SARS-CoV-2 nucleic acid testing, and a positive result upon re-testing). The husband’s chest CT showed multiple GGOs and consolidation near the pleura of the right lung, while the wife’s chest CT showed patchy shadows near the pleura in the right lung (Fig. 3e & f). However, their son had no symptoms and normal WBC and lymphocyte counts with multiple GGOs and patchy shadows in his left lung (Fig. 3g). After three negative viral nucleic testing of throat swabs or sputum, he was diagnosed COVID-19-negative. One familial cluster occurred in the COVID-19-negative group (father and son). Both were diagnosed with *Mycoplasma pneumonia* after multiplex PCR nucleic acid testing with hyperpyrexia and cough.

**Fig. 4:**
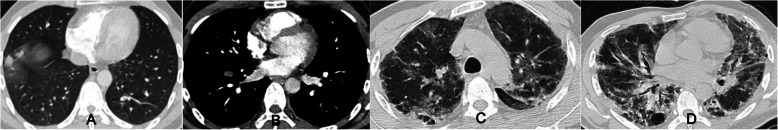
Chest computed tomography (CT) images of **a & b** Pulmonary embolism of arteries in the anterior basal segment of the right lower lung (indicated by the arrows); **c & d** dermatomyositis with pulmonary involvement

There were two noninfectious cases in COVID-19-negative group. One is 28-year-old previously healthy male patient who was diagnosed suspected case of COVID-19 due to cough, fever, increasing chest tightness gradually and GGOs and consolidation images in anterior basal segment of right lower lung of chest CT. Finally, Deep venous ultrasound showed right femoral vein thrombosis and computed tomography pulmonary angiogram (CTPA) showed multiple pulmonary embolisms in both lungs (Fig. 4a & b). His medical history showed often long-term sedentary position in last 3 months for a test, and intermittent pain in his right lower extremity. The other suspected case had cough, fever, dyspnea and rashes symptoms with interstitial abnormalities in his both lungs (Fig. 4c & d). This patient was eventually diagnosed as dermatomyositis with pulmonary involvement through testing of the spectrum of idiopathic inflammatory myopathies as.

## Discussion

As cases of COVID-19 increase in number worldwide, clinicians are struggling to diagnose new cases quickly enough to implement appropriate isolation measures. This is particularly difficult given how closely symptoms of COVID-19 match other common viral respiratory infections, including influenza. The aim of this study was to summarize the diagnostic features of suspected cases of COVID-19 in our hospital to help improve differential diagnosis, reduce misdiagnosis in future. Results of our study suggest that pneumonia in COVID-19 patients and pneumonia caused by other pathogens (eg, influenza viruses, adenovirus, and Mycoplasma) are difficult to distinguish based on their clinical manifestations, which included fever, cough, and fatigue in our study. Rarer clinical manifestations, such as expectoration, sore throat, intolerance of cold, shivering, chest tightness, dyspnea, palpitation, and diarrhea, were also common to both COVID-19 and other respiratory pathogens, which was similar to the results from previous studies [8, 9].

Routine blood tests of COVID-19-positive patients showed that the WBC count was reduced, inversely correlating with the severity of fever, instead of COVID-19-negative patients. This may contribute to the differential diagnosis of suspected cases. Approximately half (47.1%) of COVID-19-positive patients had reduced lymphocyte count; therefore, a reduced lymphocyte count in suspected cases of COVID-19 suggests the possibility of COVID-19. C-reactive protein of the COVID-19-positive patients was elevated, but was not significant for differential diagnosis. Most COVID-19-positive cases had bilateral pulmonary involvement with GGOs, multiple patchy shadows, and consolidation in their chest upon HRCT imaging, which may be helpful for differential diagnosis. However, these Chest HRCT imaging including patchy shadows, nodular shadows and grid-like also seen in the pneumonia caused by Influenza A virus, influenza B virus and adenovirus, consistent with the previous reports [10, 11].

Clustered occurrence is one of the epidemiological criteria for the diagnosis of COVID-19 [12, 13]. Our study also shows 6 out of 17 cases were clustered, but clustering is not unique to COVID-19. *Mycoplasma pneumonia* can also occur in cluster with GGOs in chest CT. In this study, the 7 patients diagnosed with *Mycoplasma pneumonia* had a mean age of 29.5 years. Three of the patients were younger than 20 years old. Bronchial wall thickening, characteristic change of *Mycoplasma pneumonia*, in the chest HRCT of young adults may help distinguish *Mycoplasma pneumonia* from COVID-19.

Additionally, we recommend performing multiplex PCR nucleic acid testing using throat swabs or sputum. It should be noted that these results may be related to factors such as sampling quality, specimen preservation, and different nasopharyngeal virus concentrations at different stages of the disease [14]. Using multiplex PCR, we distinguish influenza A virus, influenza B virus, Adenovirus, Chlamydia pneumoniae, *Mycoplasma pneumonia*e and so on from suspected cases easily. In this study, no patient with COVID-19 was found co-infection with other respiratory pathogen(s). Recent report showed that rate of co-infection between SARS-CoV-2 and other respiratory pathogens reached 20.7% in northern California, USA. So, testing of SARS-CoV-2 should been done for patients with non–SARS-CoV-2 respiratory pathogens in high incidence of COVID-19 region [15]. The detection of non–SARS-CoV-2 respiratory pathogens by multiplex PCR may help to assess individual the risk of COVID-19 in areas of low transmission [16]. Because of the highly infectious nature of SARS-CoV2, the suspected COVID-19 cases were all isolated and monitored in a single-person single-room isolation ward. Although communication with healthcare professionals was limited, a detailed medical history should not be neglected. Therefore, it is important to remain open to all causes of lung pathology, including non-infectious causes like pulmonary embolism. For suspected COVID-19 cases, a comprehensive multidisciplinary collaborative diagnosis and treatment (MDT) mechanism should be established. Relevant departments including respiratory medicine, infectious diseases, and radiology should collaborate closely when COVID-19 is suspected to avoid misdiagnosis. Positive result of SARS-CoV-2 nucleic acid testing remains the gold standard for the diagnosis of COVID-19. However, highly suspicious cases with false negative viral nucleic acid testing results should have chest CT and consecutive viral nucleic acid testing in different specimens collected from multiple regions of the body (eg, sputum, throat swabs, blood, urine, and feces) [17]. These patients should also have the tests of serum SARS-CoV-2 specific-IgM and IgG antibodies [18] to improve the diagnosis rate.

This study has some limitations. Because COVID-19 was managed as Class A infectious disease, this study only performed routine blood tests, C-reactive protein, chest HRCT, throat swab SARS-CoV-2 nucleic acid testing, but not blood biochemical tests in the patients. As a result, we cannot comment on co-morbidities in our population. Additionally, the number of cases in this study was limited by the fact that COVID-19 is an emerging disease, and our findings need to be further verified by a large-scale study.

## Conclusions

The clinical characteristics of patients with confirmed diagnosis of COVID-19 were similar to those negative cases. However, WBC count inversely correlated with the severity of fever, GGOs, multiple patchy shadows, and consolidation in chest HRCT and clustered infection are common but not specific features in the confirmed COVID-19 group. Multiplex PCR nucleic acid testing helped differential diagnosis for suspected COVID-19 cases.

## Data Availability

The datasets of the current study are not publicly available due individual privacy of patients could be involved, are available from the corresponding author on request.

## Abbreviations

COVID-19: Coronavirus disease 2019
CT: Computed tomography
GGOs: Ground-glass opacities
HRCT: High-resolution computed tomography
MDT: Multidisciplinary collaborative diagnosis and treatment
NA: Not applicable
RT-PCR: Reverse transcriptase polymerase chain reaction
SARS-CoV: Coronaviruses
SARS-CoV-2: Severe acute respiratory syndrome coronavirus 2
WBC: White blood cell
WHO: World Health Organization
